# Novel Erythrocyte-like Graphene Microspheres with High Quality and Mass Production Capability via Electrospray Assisted Self-Assembly

**DOI:** 10.1038/srep03327

**Published:** 2013-11-25

**Authors:** Yayang Tian, Guan Wu, Xike Tian, Xiaoming Tao, Wei Chen

**Affiliations:** 1 i-Lab, Suzhou Institute of Nano-Tech and Nano-Bionics, Chinese Academy of Sciences, Suzhou, 215123 (P. R. China); 2 Institute of Textiles and Clothing, The Hong Kong Polytechnic University, Hong Kong SAR; 3 Faculty of Material Science and Chemical Engineering, China University of Geosciences, Wuhan, 430074 (P. R. China)

## Abstract

We report for the first time a novel erythrocyte-like graphene microsphere (ELGMs) which can be produced with high quality and mass production capability via electrospray assisted self-assembly. Through simple electrospray treatment of GO suspension into coagulation bath followed by chemical reduction, large quantity of ELGMs with uniform morphology and size can be obtained with production rate of around 2.4 mg/h. Compared with other 3D structures, the ELGMs have a very interesting structural characteristic of perfect exterior doughnut shape and interior porous network. Accordingly, the as-prepared porous ELGMs exhibit excellent capability for fast and recyclable removal of oil and toxic organic solvents from water, reaching up to 216 times of its weight in absorption efficiency, which is tens of times higher than that of conventional sorbent materials. It is strongly believed that the novel hierarchical graphene structures and synergy among different techniques will lead to more future advances in graphene applications.

Graphene, a two dimensional (2D) monolayer of sp2-hybridized carbon atoms, has attracted great attention recently due to its superior electronic, thermal and mechanical properties^1^. These unique properties make graphene sheet a promising candidate as structure and functional component in catalysis^2^,^3^, energy storage^4^,^5^,^6^ and environment remediation applications^7^,^8^. Integration of graphene sheets, the 2D nanoscale building blocks, into three dimensional (3D) assemblies has proved to be an essential method to explore advanced properties of the individual graphene for macroscopic applications. In order to meet demands of the above applications, chemically derived graphene oxide (GO) is regarded as an ideal source for mass production of graphene owing to its easy preparation and processing^9^,^10^,^11^,^12^. Several approaches, such as layer-by-layer deposition^13^, flow-directed assembly^14^, template-directed method^15^, and leavening strategy^16^, have been so far developed to assembly graphene into layered and porous 3D macroscopic architectures. For instance, 3D graphene foams and sponges exhibit unique flexible network, high specific surface area, and outstanding electrical and mechanical properties, which enable significant progress in developing flexible electronics and energy-related materials^17^. It is of great scientific and technological importance to assembly such 2D building blocks into organized hierarchical structures. Recent attentions have been focused on the possibility of various graphene 3D structures and assembly avenues, such as hollow graphene oxide spheres by water/oil emulsion^18^, hollow graphene spheres by hydrothermal method^19^, and hollow graphene capsules by capillary molding^20^. It is quite believed that the novel hierarchical graphene structures and synergy among different techniques will lead to more future advances in graphene applications.

Herein, we report for the first time a novel erythrocyte-like graphene microspheres (ELGMs) which can be produced with high quality and mass production capability via electrospray assisted self-assembly. Through simple electrospray treatment of GO suspension into coagulation bath followed by chemical reduction, large quantity of ELGMs with uniform morphology and size can be obtained with production rate of around 2.4 mg/h. Compared with other 3D structures, the ELGMs have a very interesting structural characteristic of perfect exterior doughnut shape and interior porous network. Diameters of the ELGMs are in range of 70–200 μm which can be easily tuned mainly with concentrations of GO suspension and surfactant in coagulation bath. Compared with those already existed methods, electrospray assisted self-assembly is introduced to be a more effective and easy-handle manufacturing approach to produce novel graphene functional materials in a continuous, controllable and large-scale manner. Accordingly, the as-prepared porous ELGMs exhibit excellent capability for fast and recyclable removal of oil and toxic organic solvents from water, including diesel oil, chloroform, petroleum ether and etc, reaching up to 216 times of its weight in absorption efficiency, which is tens of times higher than that of conventional sorbent materials. Details are reported as follows.

## Results

### Electrospray assisted self-assembly of ELGMs

**Figure 1:**
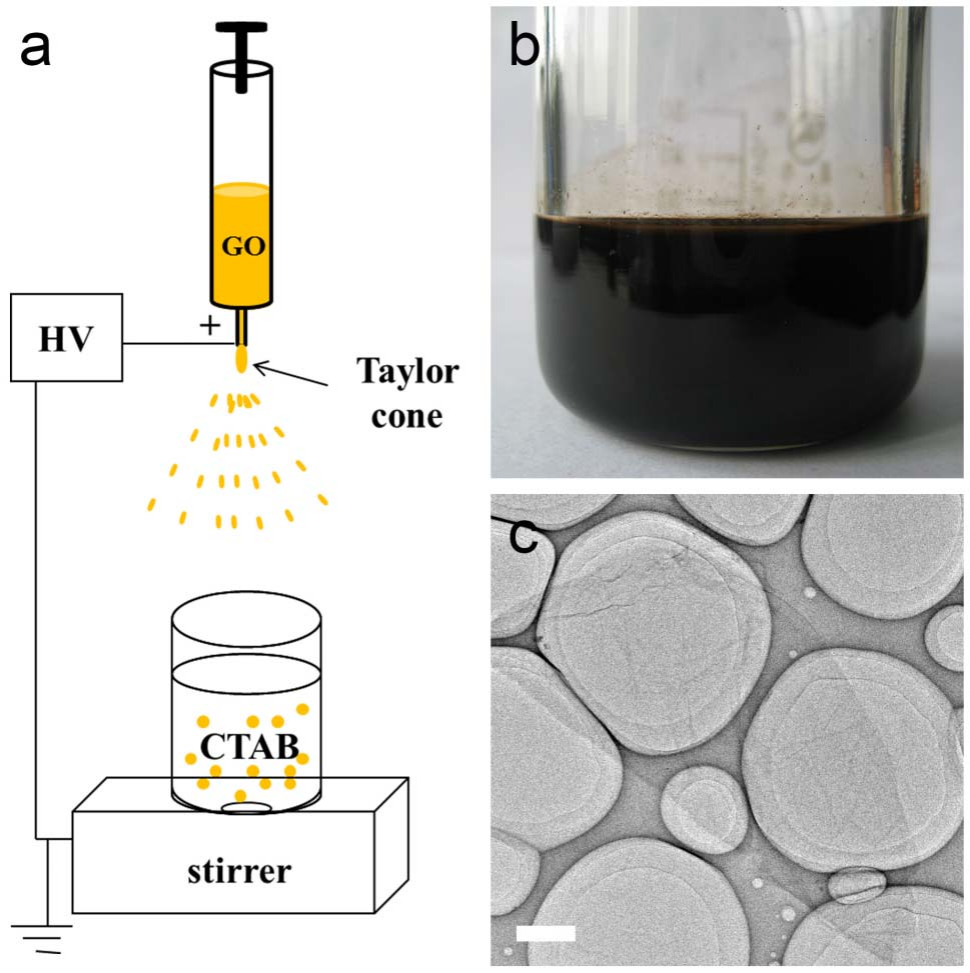
Schematic of the electrospray and GO suspension solution. (a) Schematic of the electrospray assisted self-assembly process. (b) The photograph of 20 ml GO aqueous dispersion at a concentration of 12.5 mg/ml. (c) TEM image of the individual GO sheet (scale bar 1 μm).

Figure 1a illustrates the schematic of electrospray assisted GO self-assembly for fabricating novel ELGMs. The set-up is constructed in a commercialized electrospin apparatus (see Supplementary Figure S1a), consisting of a high voltage power, spinning GO solution in a syringe, and a grounded collector (e.g. a coagulation bath). The syringe with needle was connected to a positive high voltage power, while the collector was grounded. Process conditions including applied voltage, flow rate, and stirring rate can be easily and accurately adjusted. When a sufficiently high voltage is applied, the body of the liquid becomes charged, and electrostatic repulsion counteracts the surface tension and the droplet is stretched; at a critical point a stream of liquid erupts from the surface. This point of eruption is known as the Taylor cone. Those erupted droplets were then collected into the cetyltrimethylammonium bromide (CTAB) coagulation bath to form GO microspheres in large scale. (see Supplementary Figure S1b). Here the GO solution suspension with concentration up to 12.5 mg/mL was prepared from natural graphite flakes (< 30 μm) by a modified Hummers method reported previously, and had stable solubility in water because of their plentiful oxygen-containing functional groups on GO sheets (Figure 1b). TEM image shows the typical size of individual GO sheets is around 2 μm (Figure 1c). In order to get uniform, large-scale ELGMs by electrospinning, processing conditions including concentration of GO suspension and CTAB in coagulation bath, the applied voltage, flow rate, and stirring rate, are systematically investigated and optimized.

### Characterization and controllable preparation of erythrocyte-like graphene oxide microspheres and ELGMs

**Figure 2:**
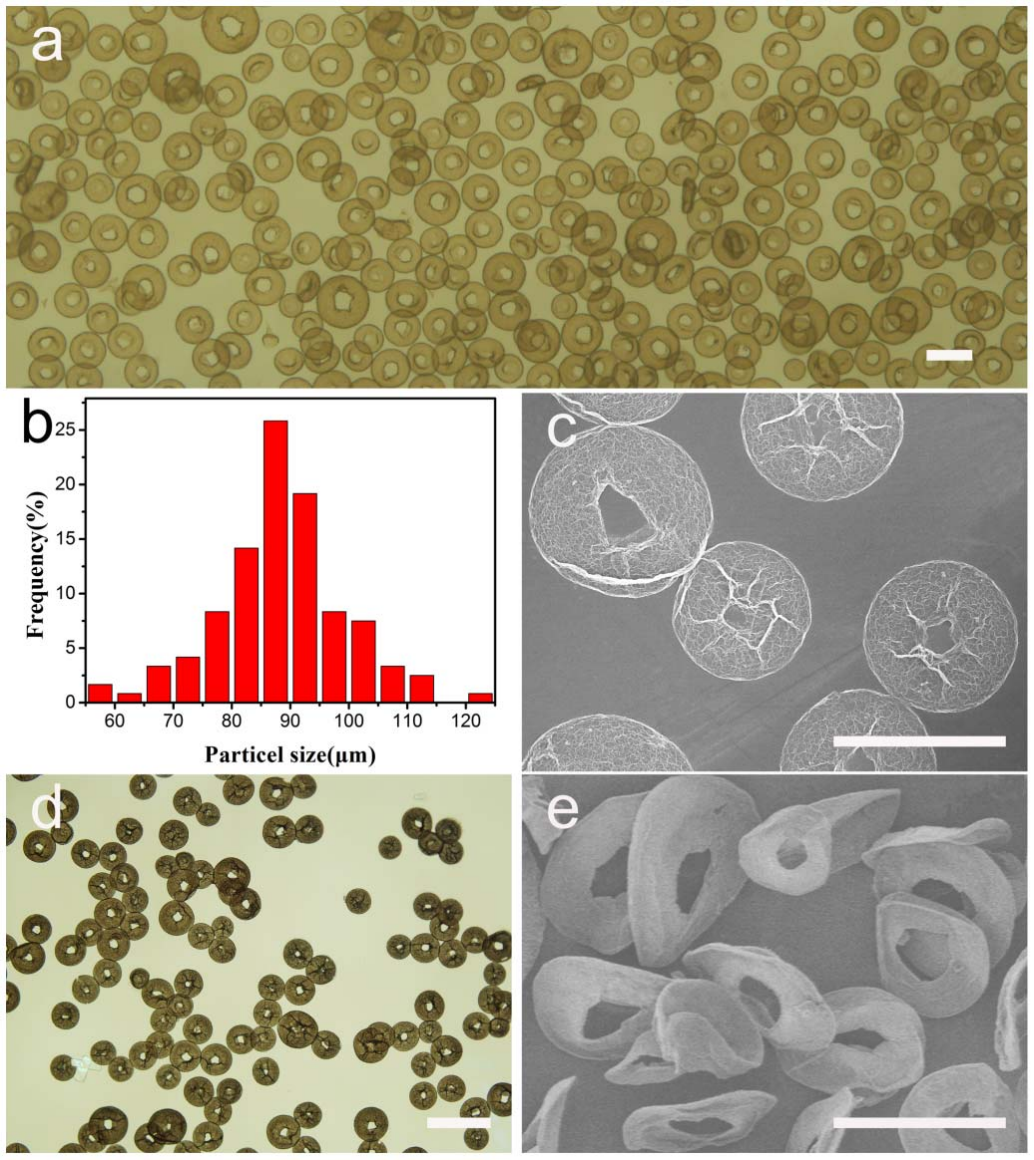
Microstructures of microspheres. A general Optical microscope view of the erythrocyte-like GO microspheres in CTAB solution by electrospray of the 12.5 mg/ml GO suspension in a coagulation bath of 0.75 mg/ml CTAB solution (scale bar 200 μm); (b) Size distribution of erythrocyte-like GO microspheres. (c) SEM image of the erythrocyte-like GO microspheres (scale bar 100 μm). (d) Optical microscope image of erythrocyte-like GO microspheres after drying (scale bar 200 μm). (e) SEM image of erythrocyte-like graphene microspheres reduced by hydrazine hydrate (scale bar 100 μm).

**Figure 3:**
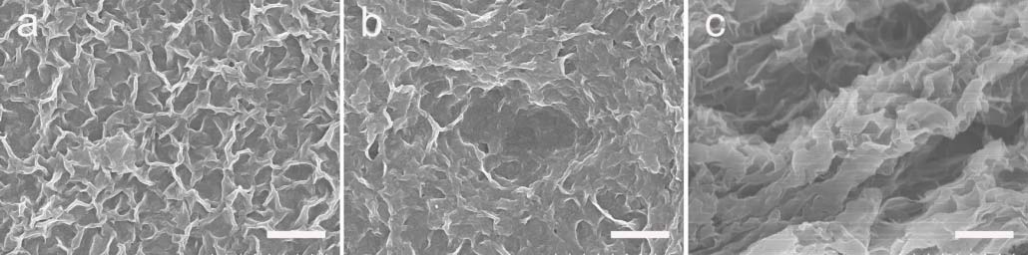
SEM images of the inner surface (a), outer surface (b), and cross-section (c) for erythrocyte-like GO microspheres (scale bar 100 μm).

Figure 2a shows the general optical microscopy image of doughnut-like GO microspheres by electrospray of 12.5 mg/ml GO suspension in a coagulation bath of 0.75 mg/ml CTAB solution at an applied voltage of 9 KV, flow rate of 0.05 mm/min and stirring rate of 100 rpm. The erythrocyte-like graphene oxide microspheres have a very interesting structural characteristic of perfect exterior doughnut shape and interior porous network (Figure 3). Massive GO microspheres with uniform morphology and size are present. From statistic size distribution of these spheres as shown in Figure 2b, mean diameter is around 90 μm. SEM image (Figure 2c) further reveals the interior porous structure of each microsphere. The dried microspheres keep the same morphology as they are in wet state, but show a little shrinkage in outer diameter and expansion in inner diameter (Figure 2d). In order to get the ELGMs, GO microspheres were chemically reduced by using hydrazine hydrate. It can be found in Figure 2e that these reduced GO microspheres, which are called ELGMs, can still maintain the doughnut morphology and porous microstructure. To study the mechanical stability, the erythrocyte-like GO microspheres overcame the centripetal force and remained the morphology under vigorous stirring with 700 rpm (see Supplementary Figure S2a). The ELGMs have also been verified to have good mechanical stability during repeated drying and washing process. Image data confirmed that the ELGMs appeared to preserve their shapes and sizes (see Supplementary Figure S2b), but looked a little wizened when being subjected to freeze drying and SEM observation under vacuum as shown in Figure 2e. In comparison with the FT-IR spectra of erythrocyte-like graphene oxide microspheres and ELGMs in Figure S3. For the erythrocyte-like graphene oxide microspheres, some bands were observed in the region 2800–3000, 1450–1500 and 2980 cm^−1^ and attributed to CTAB molecules^21^. The broad peak at 3000–3600 cm^−1^ and band centered at 1622 cm^−1^ corresponds to the stretching mode of O-H. The physically adsorbed H_2_O molecules also contribute to this broad peak^22^. After reduced to erythrocyte-like graphene microspheres and repeated water washing process, the bands of CTAB were disappeared, indicating the complete removal of CTAB molecules. CTAB molecules are removed from the microspheres due to the significant decrease of electrostatic attractions followed with GO reduced to graphene sheets. After the CTAB was removed, the structures of ELGMs were still stable (Figure 2e).

**Figure 4:**
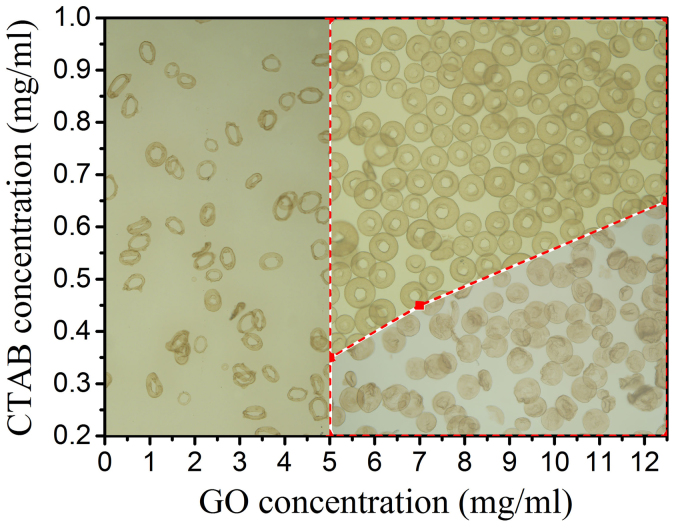
Controllable preparation of erythrocyte-like GO microspheres. Schematic depiction of the effect of GO and CTAB concentration on morphology of erythrocyte-like GO microspheres.

Herein, most remarkable of the electrospray assisted self-assembly is that microspheres structure can be easily adjustable and controllable under solution conditions and electrospin parameters. In our electrospray method, CTAB solution is chosen as the coagulation bath due to its positive charged ammonium ion which may play a surprising role in the assembly of the GO droplet to microsphere^23^. The solution conditions including concentration of GO suspension and CTAB in coagulation bath, are found to be the most crucial parameters in fabricating ELGMs, which has been distinctly verified in the evolution process of microspheres under different concentrations of GO and CTAB solutions as shown in Figure 4. When GO suspension has lower concentrations (less than 5 mg/mL), only elastic–shaped microspheres are formed (see Supplementary Figure S4a). When the GO concentration is increased to the range of 5.0–12.5 mg/mL, large scale erythrocyte-like microspheres can be obtained at relatively high CTAB concentration (e.g. in the range of 0.35–1.0 mg/mL) (see Supplementary Figure S4b), but round wrinkled microspheres are formed at lower CTAB concentrations (see Supplementary Figure S4c), indicating that both GO and CTAB concentrations predominantly influence the microsphere shape. Besides, electrospin parameters including applied voltage, flow rate and stirring rate, are also investigated (see Supplementary Figure S5–S7). What they influence is mainly the size and uniformity of erythrocyte-like microspheres. The microsphere diameter decreased as the applied voltage increased. At a high-applied voltage, however, charge acceleration caused the spray solution not to be separated and thus the microsphere diameter increased. As the flow rate of GO suspension increased, the microsphere diameter slightly increased. As the stirring rate of coagulation bath increased, the diameter of the microspheres decreased.

### Electrospray assisted self-assembly mechanism of erythrocyte-like graphene oxide microspheres

**Figure 5:**
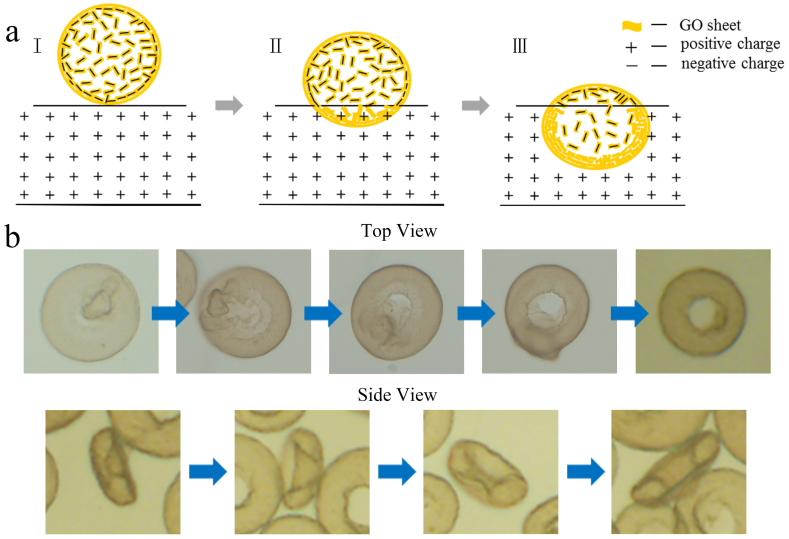
Formation mechanism of the erythrocyte-like GO microspheres. (a) Schematic illustration of electrospray process of GO droplet into CTAB solution. (b) Optical microscope images showing the assembly mechanism of erythrocyte-like GO microspheres. The Optical microscope images were obtained steady states under various CTAB concentrations.

Based on the above experimental studies, formation mechanism of the erythrocyte-like microspheres is proposed in Figure 5a and 5b. Figure 5a illustrates the electrospray and assembly process into three stages. In stage I, at Taylor cone the GO liquid is erupted into many small droplets in which plenty of negative charged GO sheets are included, and will be collected by a coagulation bath where CTAB molecules with positive charged ammonium ions are incorporated. Next when droplets meet CTAB solution, electrostatic attractions between CTAB molecules and GO sheets take place firstly at the bottom of a GO droplet as shown in stage II. Then CTAB molecules diffuse into the droplet, and GO sheets closer to the droplet surface begin to coagulate and stack. As the droplets continue falling into CTAB solution, the coagulated GO shell gradually increases in thickness, forming a solid skin. In stage III, the top layer of droplet is also the last place where the interaction with CTAB takes place, leaving the thinnest and weakest GO coagulated shell. In the case of lower CTAB concentration, stable GO microspheres with wrinkles can be usually observed (Figure 4). While in the case of higher CTAB concentration, such as in the range of 0.65–1.0 mg/mL, more CTAB molecules continue to diffuse into the interior of microsphere from the thinnest and weakest top layer. Therefore, the interactions between CTAB and residual GO sheets within microspheres weaken the mechanical strength of the outer layer of the microspheres, which caused the structural change on the top layer of spheres^24^. That is, the top layer collapsed, and the microspheres finally turned into erythrocyte-like GO microspheres. Figure 5b further illustrates the formation mechanism by using experimental images observed under various solution conditions. We cannot capture the intermediate states in a constant concentration of CTAB, but can use the obtained steady states under various CTAB concentrations to explain the mechanism.

### Various oils and toxic organic solvents adsorption and desorption in ELGMs

**Figure 6:**
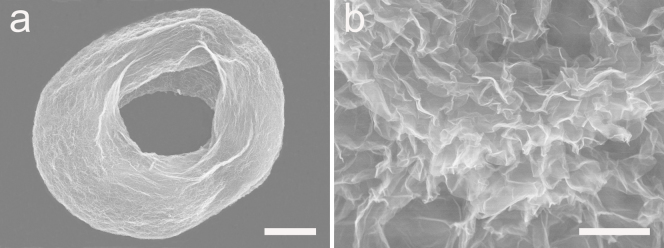
ELGMs reduced by Hydrazine Hydrate. (a) SEM image of a single ELGM (scale bar 25 μm). (b) Higher magnification SEM image of the ELGMs (scale bar 1 μm).

**Figure 7:**
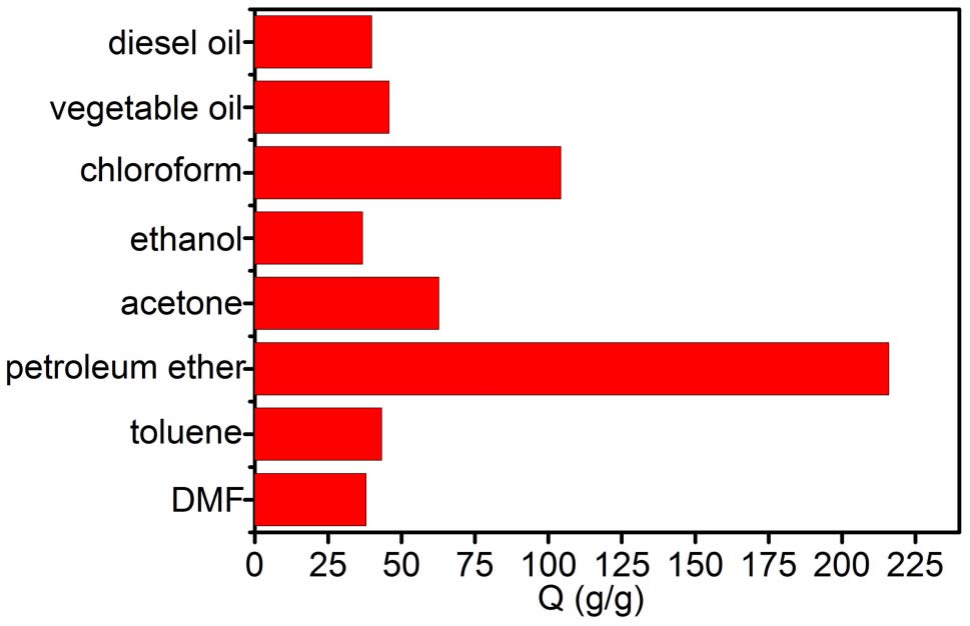
Absorption capacities of ELGMs. Absorption capacities (Q) of ELGMs measured for a range of oils and organic solvents.

**Figure 8:**
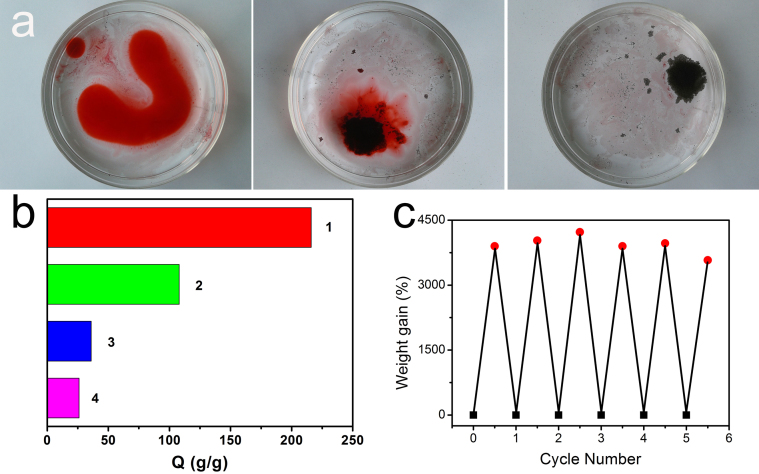
Absorption characterization of ELGMs. (a) Absorption process of petroleum ether (stained with oil red dye) on water by the ELGMs within 30 s. (b) The diagram of absorption capacities of various absorbents for petroleum ether. The numbers represent the ELGMs in this study (1), activated carbon (2), conventional graphene film (3) and carbon black (4). (c) The absorption recyclability of ELGMs over several cycles.

It has been widely reported that those porous microspheres have several structural features and properties that could make them attractive for oil adsorption and recovery. For example, they are lightweight powders with large free volumes, which are spreadable over large areas to collect oils. For that reason, erythrocyte-like GO microspheres are further reduced by using hydrazine hydrate to get the ELGMs. After being reduced, CTAB molecules have left the microspheres due to significant decrease of electrostatic attractions among the graphene sheets^25^. Figure 6a and b give the typical doughnut shape and interior porous structure of an individual ELGM. Specific surface areas of the ELGMs is measured to be 6.167 m^2^ g^−1^ by Brunauer–Emmett–Teller (BET) method, and pore size distributions for ELGMs are in the range of 10–50 nm (see Supplementary Figure S8). To evaluate the adsorption performance, adsorption capacity (Q) is used, which is defined by the ratio of the final weight after full absorption to the initial weight of ELGMs. It can be found that the ELGMs exhibit excellent capability for removal of oil and toxic organic solvents from water, including DMF, toluene, acetone, diesel oil, chloroform, petroleum ether and etc., reaching from 36 up to 216 times of its weight in adsorption efficiency as shown in Figure 7, which is tens of times higher than that of conventional sorbent materials such as natural absorbers^26^, and polymers^27^,^28^. It can be found that the adsorption capacity of ELGMs fluctuated with different organic solvents and oils. The adsorption depends on not only density but also polarity and viscosity of the chemical molecule, which has been widely recognized in the organic chemical-carbon nanotube interaction^29^. Therefore, the high adsorption selectivity of ELGMs for the nonpolar petroleum ether is dominantly attributed to the hydrophobic π-π stacking of the reduced graphene oxide^30^. The relative lower adsorption capacity for other solvents and oils, such as DMF, ethanol and vegetable oil, may ascribe to their strong polarity and viscosity. Figure 8a shows that 648 mg petroleum ether (colored with an oil red dye) on water is adsorbed completely and quickly by 3 mg ELGMs powders within 30 s, indicating high adsorption efficiency with Q value of 216 g g^−1^. Compared with other sorbent materials such as carbon black, activated carbon and conventional graphene film, the Q value of ELGMs to petroleum ether is the highest as shown in Figure 8b. Pollution control and environmental protection efforts require that pollutants are not only absorbed and prevented from further harming the environment, but also properly recycled and thus reused, as they are either precious raw materials or toxic. More importantly, the absorbed organics can be easily collected and re-used by drying samples at 70°C. No combustion or structural damage occurs in heat treatments at moderate temperatures. In this way, the ELGMs can be re-used without significant loss in adsorption capacity just after the simple heat treatment (Figure 8c). Chemical extraction or vacuum treatment is necessary, instead of heat treatment, for polymeric absorbents because these are sensitive to heat, resulting in incomplete recycling of pollutants, degradation of materials, and higher costs. Therefore, although the ELGMs do not have very high surface area, they have good absorption performance for organic solvents and oils. The hydrophobic surface and strong π-π interactions between ELGMs and organics are proposed to understand the mechanism^20^,^29^. Further investigations regarding the superior performance of ELGMs for more organic solvents and oils adsorption as well as their in catalysis and energy storage applications are in progress.

## Discussion

According to the characterizations of the microstructures under various solution conditions of the erythrocyte-like GO microspheres, the formation mechanism of the microspheres is described by schematic illustrations as shown in Figure 5a and Figure 5b. Since the GO droplets contact with the positive-charged CTAB solution, as sketched in Figure 5a, the electrostatic attractions between CTAB molecules and the GO sheets take place. Initially, CTAB molecules diffuse into the droplet, and GO sheets closer to the droplet surface begin to coagulate and stack, forming a solid skin at the bottom of the GO droplet. A thinnest and weakest GO coagulated shell on the top layer of droplet occurs quickly once the droplet is immersed in the coagulation solution. Then, more CTAB molecules continue to diffuse into the interior of microsphere from the thinnest and weakest top layer. Afterwards, the interactions between CTAB and residual GO sheets within microspheres weaken the mechanical strength of the outer layer of the microspheres, which caused the structural change on the top layer of spheres, finally turned into erythrocyte-like GO microspheres. In this way, the positive-charged CTAB as the coagulation solution induces the assembly of the erythrocyte-like GO microspheres in a high-order manner.

In conclusion, high quality and mass production capability of novel erythrocyte-like graphene oxide and graphene microspheres via electrospray assisted self-assembly have been verified. The most remarkable feature of this technique is that the microsphere structure can be easily adjustable and controllable under solution conditions and electrospin parameters. Accordingly, the as-prepared porous ELGMs exhibit excellent capability for fast and recyclable removal of oil and toxic organic solvents from water, including diesel oil, chloroform, petroleum ether and etc, reaching up to 216 times of its weight in absorption efficiency, which is tens of times higher than that of conventional sorbent materials. Further integration of various guests functional materials into the novel ELGMs host are highly expected to endow more new or enhanced properties, which will definitely lay the solid foundation for exploring potential applications in environment remediation, energy storage and catalysts etc.

## Methods

### Fabrication of GO microspheres

For a detailed description of the materials used and preparation of GO see the Supporting Information. GO microsphere was prepared by electrospray. The 12.5 mg/mL of GO dopes were pumped through a plastic syringe connected with a metal syringe needle by an injection pump. The application of a high voltage (9 kV) to the metal syringe needle enabled the generation of microspheres, which were collected on the CTAB coagulation bath.

### Chemical Reduction of GO microspheres

GO microspheres were chemically reduced by the hydrazine hydrate at 90°C for 12 h, followed by freeze-drying.

### Uptake studies of ELGMs

Before investigating the absorption behavior for petroleum ether, we labeled the petroleum ether with Oil Red dye. In the case of the oil and toxic organic solvents, up to the quality of the oil and toxic organic solvents is completely sorbed into 1 g of the ELGMs.

### Characterization

TEM, FESEM, and High-resolution optical microscope images were recorded using a FEI Tecnai G2 F20 S-Twin 200 KV, Quanta 400 FEG and Olympus BX51 separately. FTIR spectra were recorded on Nicolet 6700 IR spectrometer by using KBr pellets.

## Author Contributions

Y.Y.T. planned and performed the experiments, collected and analyzed the data, and wrote the paper. W.C. supervised the project, and conceived the experiments, analyzed the results and wrote the paper. G.W. helped with synthesis of the materials and collected the data. Y.Y.T. was with suggestion from X.K.T. and X.M.T. All authors discussed the results and commented on the manuscript.

## Supplementary Material

Supplementary Information Supplementary Information

## References

[b1] Allen M. J., Tung V. C. & Kaner R. B. Honeycomb Carbon: A Review of Graphene. Chem. Rev. 110, 132–145 (2010). 19610631 10.1021/cr900070d

[b2] Trancik J. E., Barton S. C. & Hone J. Transparent and Catalytic Carbon Nanotube Films. Nano Lett. 8, 982–987 (2008). 18302327 10.1021/nl071945i

[b3] Wang X., Zhi L. & Mullen J. K. Transparent, Conductive Graphene Electrodes for Dye-Sensitized Solar Cells. Nano Lett. 8, 323–327 (2008). 18069877 10.1021/nl072838r

[b4] Futaba D. N. *et al.* Shape-engineerable and highly densely packed single-walled carbon nanotubes and their application as super-capacitor electrodes. Nat. Mater. 5, 987–994 (2006). 17128258 10.1038/nmat1782

[b5] Liu J. H. & Liu X. W. Two-Dimensional Nanoarchitectures for Lithium Storage. Adv. Mater. 24, 4097–4111 (2013). 10.1002/adma.201104993 22504798

[b6] Lee S. T. *et al.* Facile Synthesis of a Large Quantity of Graphene by Chemical Vapor Deposition: an Advanced Catalyst Carrier. Adv. Mater. 24, 2491–2495 (2012). 22489026 10.1002/adma.201200480

[b7] Bi H. C. & Ruoff R. S. Spongy Graphene as a Highly Efficient and Recyclable Sorbent for Oils and Organic Solvents. Adv. Funct. Mater. 22, 4421–4425 (2012).

[b8] Sun H. Y., Xu Z. & Gao C. Multifunctional, Ultra-Flyweight, Synergistically Assembled Carbon Aerogels. Adv. Mater. 25, 2554–2560 (2013). 23418099 10.1002/adma.201204576

[b9] Chen C. *et al.* Self-Assembled Free-Standing Graphite Oxide Membrane. Adv. Mater. 21, 3007–3011 (2009).

[b10] Cote L. J., Kim F. & Huang J. X. Langmuir-Blodgett Assembly of Graphite Oxide Single Layers. J. Am. Chem. Soc. 131, 1043–1049 (2009). 18939796 10.1021/ja806262m

[b11] Dikin D. A. *et al.* Preparation and characterization of graphene oxide paper. Nature 448, 457–460 (2007). 17653188 10.1038/nature06016

[b12] Xu Y. F. *et al.* A Graphene Hybrid Material Covalently Functionalized with Porphyrin: Synthesis and Optical Limiting Property. Adv. Mater. 21, 1275–1279 (2009).

[b13] Chen W. *et al.* Graphene-Stabilized Silver Nanoparticle Electrochemical Electrode for Actuator Design. Adv. Mater. 25, 1270–1274 (2013). 23184560 10.1002/adma.201203655

[b14] Yang X. W., Zhu J. W., Qiu L. & Li D. Bioinspired Effective Prevention of Restacking in Multilayered Graphene Films: Towards the Next Generation of High-Performance Supercapacitors. Adv. Mater. 23, 2833–2838 (2011). 21557338 10.1002/adma.201100261

[b15] Vickery J. L., Patil A. J. & Mann S. Fabrication of Graphene–Polymer Nanocomposites With Higher-Order Three-Dimensional Architectures. Adv. Mater. 21, 2180–2184 (2009).

[b16] Chen X. D. *et al.* A Leavening Strategy to Prepare Reduced Graphene Oxide Foams. Adv. Mater. 24, 4144–4150 (2012). 22544807 10.1002/adma.201200197

[b17] Yin S. Y., Niu Z. Q. & Chen X. D. Assembly of Graphene Sheets into 3D Macroscopic Structures. Small 8, 2458–2463 (2012). 22619180 10.1002/smll.201102614

[b18] Guo P., Song H. H. & Chen X. H. Hollow graphene oxide spheres self-assembled by W/O emulsion. J. Mater. Chem. 20, 4867–4874 (2010).

[b19] Wang Y. M. *et al.* Hollow graphene spheres self-assembled from graphene oxide sheets by a one-step hydrothermal process. Carbon 56, 389–391 (2013).

[b20] Huang J. X. *et al.* Oil absorbing graphene capsules by capillary molding. Chem. Commun. 48, 5968–5970 (2012). 10.1039/c2cc32049e 22569878

[b21] King H. S. & Kung Kim F. H. Fourier Transform Infrared Spectroscopic Study of the Adsorption of Cetyltrimethylammonium Bromide and Cetylpyridinium Chloride on Silica. Langmuir. 9, 263–267 (1993).

[b22] Li G. J. & Kawi S. Synthesis, characterization and sensing application of novel semiconductor oxides. Talanta. 45, 759–766 (1998). 18967059 10.1016/s0039-9140(97)00295-6

[b23] Liang Y. Y., Wu D. Q., Feng X. L. & Mullen K. Dispersion of Graphene Sheets in Organic Solvent Supported by Ionic Interactions. Adv. Mater. 21, 1679–1683 (2009).

[b24] Yang J. *et al.* The Preparation and Forming Mechanism of the Red Blood Cell-Shaped Microspheres via Electrospraying. J. Appl. Polym. Sci. 122, 2552–2556 (2011).

[b25] Cong H.-P., Ren X.-C., Wang P. & Yu S.-H. Wet-spinning assembly of continuous, neat, and macroscopic graphene fibers. Sci. Rep. 2, 613; 10.1038/srep00613 (2012). 22937222 10.1038/srep00613 PMC3430881

[b26] Bastani D., Safekordi A. A., Alihosseini A. & Taghikhani V. Study of oil sorption by expanded perlite at 298.15 K. Sep. Purif. Technol. 52, 295–300 (2006).

[b27] Bayat A., Aghamiri S. F., Moheb A. & Vakili-Nezhaad G. R. Oil Spill Cleanup from Sea Water by Sorbent Materials. Chem. Eng. Technol. 28, 1525–1528 (2005).

[b28] Zhu Q., Pan Q. M. & Liu F. T. J. Facile Removal and Collection of Oils from Water Surfaces through Superhydrophobic and Superoleophilic Sponges. J. Phys. Chem. C. 115, 17464–17470 (2011).

[b29] Pan B. & Xing B. Adsorption Mechanisms of Organic Chemicals on Carbon Nanotubes. Environ. Sci. Technol. 42, 9005–9013 (2008). 19174865 10.1021/es801777n

[b30] Chen W. F. & Yan L. F. In situ self-assembly of mild chemical reduction graphene for three-dimensional architectures. Nanoscale 3, 3132–3137 (2011). 21698339 10.1039/c1nr10355e

