# Supplementary material for: Novel Erythrocyte-like Graphene Microspheres with High Quality and Mass Production Capability via Electrospray Assisted Self-Assembly
Source: Sci Rep. 2013 Nov 25;3:3327. doi: 10.1038/srep03327 (PMC3839036; doi:10.1038/srep03327)
Supplement: Supplementary Information [file srep03327-s1.pdf]

Supplementary Information

Novel Erythrocyte-like Graphene Microspheres with High Quality and Mass Production Capability via Electrospray Assisted Self-Assembly

Yayang Tian[1, 3], Guan Wu[1], Xike Tian[3], Xiaoming Tao[2] and Wei Chen*[1]

1 i-Lab, Suzhou Institute of Nano-Tech and Nano-Bionics, Chinese Academy of Sciences, Suzhou, 215123 (P. R. China), 2 Institute of Textiles and Clothing, The Hong Kong Polytechnic University, Hong Kong SAR, 3 Faculty of Material Science and Chemical Engineering, China University of Geosciences, Wuhan, 430074 (P. R. China)

DETAILED EXPERIMENTAL METHODS

Materials. Natural graphite flake (325 meshes, 99.8 %) was obtained from Sinopharm Chemical Reagent Co., Ltd. All other reagents were analytical grade and used as received.

Preparation of GO. Graphite oxide was synthesized from graphite powder (< 30 μm) by a modified Hummers method reported previously1. Graphite flakes (2 g, purity, > 99.7 %; Qingdao Chemical Reagent Co. Ltd., China) and sodium nitrate (1 g) were mixed with sulfuric acid (46 mL, 98 wt. %) in an ice bath. Potassium permanganate (6 g) was added to the mixture very slowly, to avoid the temperature exceeding 20 ℃. The reaction was then kept at 35 ± 1 ℃ for 8 h, and during this time gas was released. Deionized water (92 mL) was gradually added, bringing about violent effervescence. The temperature of the water bath was increased to 98 ℃ and maintained for 15 min to increase the degree of oxidation of the GO product. The bright-yellow suspension was diluted with deionized water (280 mL) and further treated with H_2O_2 (30 %, 6 mL). Finally,

the mixture was separated by centrifugation and washed seven times with 5 % hydrochloric acid solution until no sulfate ion. The product was then washed seven times with distilled water to remove chloride ions.

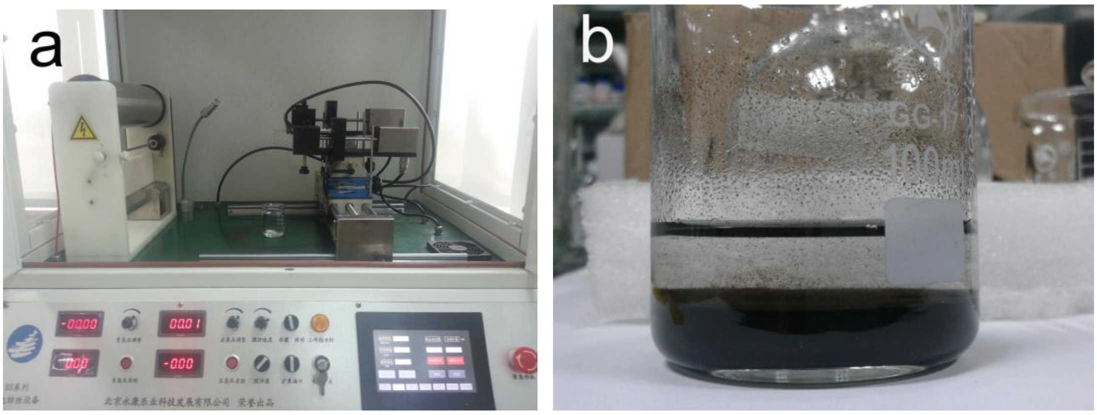

Figure S1 | a) The set-up of constructed in a commercialized electrospin apparatus. b) Photograph of erythrocyte-like GO microspheres collected by 0.75 mg/mL CTAB solution.

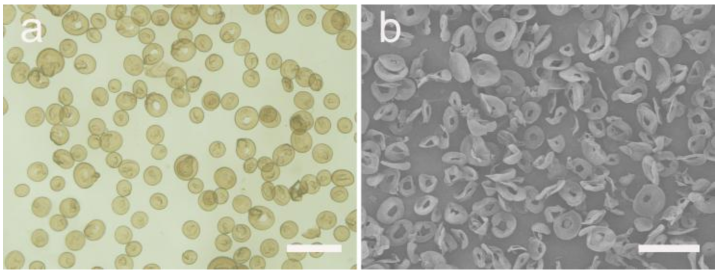

Figure S2 | a) Optical microscope image of erythrocyte-like GO microspheres after vigorous stirring (700 rpm). b) SEM image of ELGMs after repeated drying and washing process; scale bar represents 200 μm.

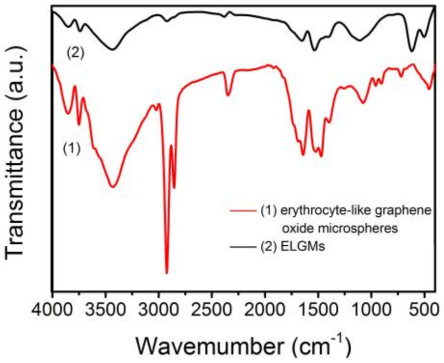

Figure S3 | The FTIR spectra of the erythrocyte-like graphene oxide microspheres and ELGMs, measured as KBr pellets.

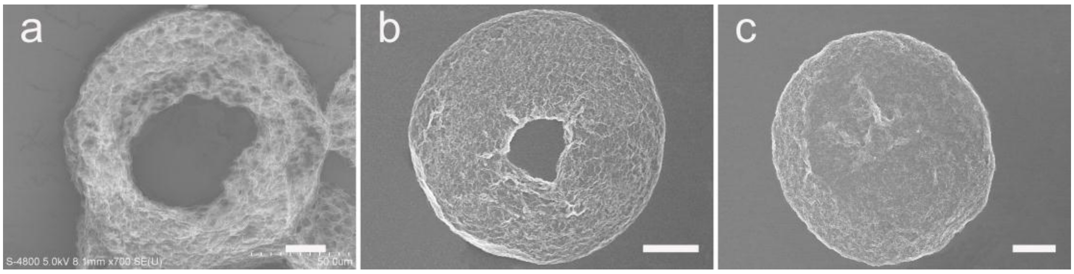

Figure S4 | SEM images of GO microspheres of different shapes, a) elastic–shaped, b) erythrocyte-like and c) round wrinkled; scale bar represents 20 µm.

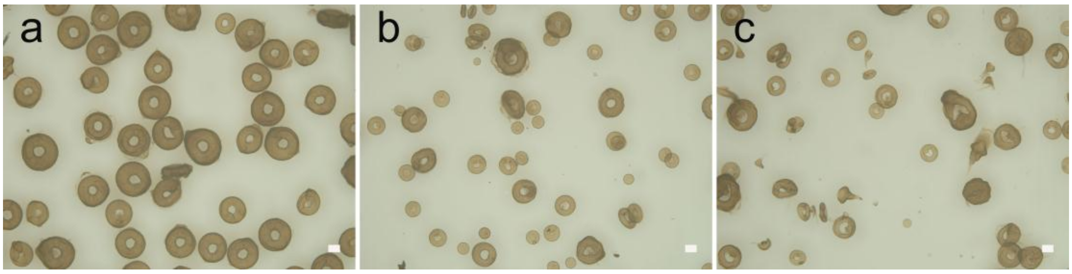

Figure S5 | Optical microscope images of erythrocyte-like GO microspheres at various applied voltage: (a) 7 KV, (b) 10.7 KV, (c) 15 KV (GO concentrations: 12.5 mg/ml; flow rate: 0.1 mm/min; stirring rate: 100 rpm; CTAB concentration: 0.5 mg/ml, scale bar 100 µm).

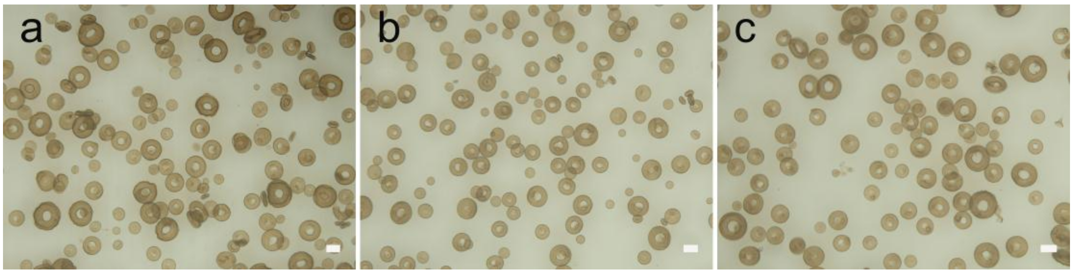

Figure S6 | Optical microscope images of GO microspheres at various flow rates: (a) 0.05 mm/min, (b) 0.01 mm/min, (c) 0.005 mm/min (GO concentrations: 12.5mg/ml; applied voltage: 7KV; stirring rate: 100 rpm; CTAB concentration: 0.5mg/ml, scale bar 100 µm).

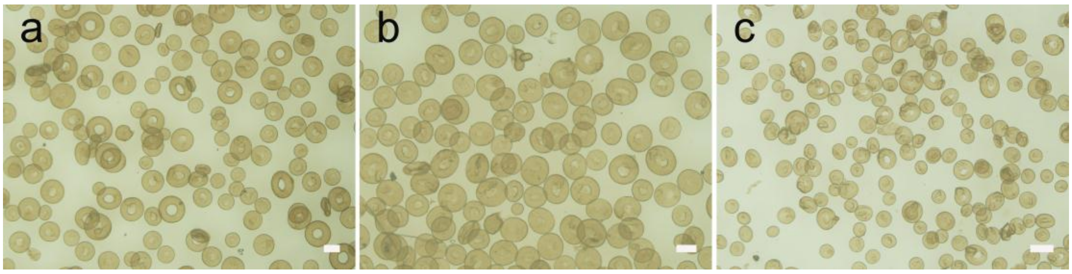

Figure S7 | Optical microscope images of GO microspheres at various stirring rate: (a) 100 rpm, (b) 400 rpm, (c) 700 rpm (GO concentrations: 12.5mg/ml; applied voltage: 7KV; flow rate: 0.1mm/min; CTAB concentration: 0.5 mg/ml, scale bar 100 µm).

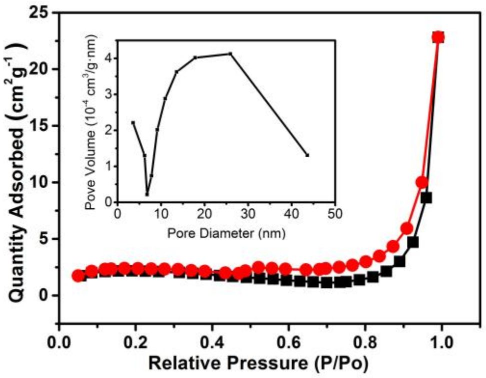

Figure S8 | Nitrogen adsorption / desorption isotherms and pore size distributions for the ELGMs.
